# Supplementary material for: Effects of Ammonia and Salinity Stress on Non-Volatile and Volatile Compounds of Ivory Shell (Babylonia areolata)
Source: Foods. 2023 Aug 25;12(17):3200. doi: 10.3390/foods12173200 (PMC10486454; doi:10.3390/foods12173200)
Supplement: Supplementary file 1 [file foods-12-03200-s001.zip › foods-2540332-supplementary.pdf]

foods

MDPI

Table S1. Volatile profiles of ivory shell muscle under different ammonia and salinity levels identified using GC-MS (Relative peak area, %)

	Name	CAS Number	Ammonia Concentrations (mg/L)					Salinity (ppt)				
			0	2.5	5	7.5	10	20	25	30	35	40
**Aliphatic Hydrocarbons**												
1	Limonene	000138-86-3	0.22±0.05b	0.55±0.08a	0.25±0.02b	0.21±0.02b	0.23±0.03b	0.30±0.02AB	0.41±0.29A	0.23±0.02AB	0.16±0.07B	0.30±0.02AB
2	1,4-Cyclooctadiene, (Z,Z)-	016327-22-3	0.73±0.12ab	0.85±0.05a	0.59±0.03bc	0.56±0.04c	0.68±0.15bc	0.66±0.02AB	0.68±0.00AB	0.79±0.09A	0.62±0.12B	0.62±0.07B
3	1,3-Cyclooctadiene	001700-10-3	0.43±0.11a	0.30±0.01bc	0.42±0.02a	0.36±0.07ab	0.24±0.06c	0.51±0.01A	0.36±0.10BC	0.47±0.09AB	0.34±0.02C	0.50±0.05A
4	Eicosane	000112-95-8	0.73±0.05a	0.52±0.05b	0.48±0.04b	0.36±0.07c	0.32±0.06c	0.11±0.01D	0.44±0.03BC	0.62±0.04A	0.51±0.06B	0.43±0.05C
5	Nonadecane	000629-92-5	0.17±0.02a	0.10±0.02b	0.17±0.00a	0.14±0.06ab	0.16±0.03ab	0.17±0.04A	0.15±0.00A	0.17±0.02A	0.17±0.02A	0.14±0.01A
6	Pentadecane, 2,6,10,14-tetramethyl-	001921-70-6	1.79±0.31a	1.43±0.09ab	0.99±0.00c	1.34±0.37bc	1.23±0.22bc	1.51±0.24B	1.36±0.18B	2.02±0.45A	1.05±0.12B	1.12±0.12B
**Alcohols**												
7	2-Decen-1-ol	022104-80-9	0.29±0.04a	0.30±0.06a	0.24±0.02a	0.26±0.02a	0.29±0.03a	0.22±0.03B	0.25±0.05B	0.33±0.01A	0.21±0.02B	0.25±0.05B
**Aldehydes**												
8	Butanal, 3-methyl-	000590-86-3	1.65±0.06b	1.70±0.12b	2.72±0.05a	2.64±0.40a	2.32±0.62a	1.85±0.49B	2.36±0.57AB	1.82±0.16B	3.20±0.78A	2.04±0.08B
9	2-Butenal, 2-methyl-, (E)-	000497-03-0	1.27±0.17cd	3.16±0.46a	2.30±0.42b	0.88±0.02d	1.88±0.62bc	5.43±2.56A	1.97±0.27B	1.27±0.17B	2.76±0.41B	1.52±0.21B
10	Hexanal	000066-25-1	25.32±4.68b	29.40±0.85ab	27.37±0.46b	32.39±3.11a	28.71±0.14ab	22.71±4.15B	28.48±1.93A	26.73±1.18AB	28.42±1.69A	25.38±2.67AB
11	2-Hexenal, (E)-	006728-26-3	1.50±0.11ab	0.46±0.07c	1.14±0.74b	2.06±0.27a	1.52±0.04ab	1.95±0.08AB	1.73±0.30AB	1.53±0.31B	1.87±0.28AB	2.04±0.23A
12	Heptanal	000111-71-7	6.84±0.12c	6.99±0.29bc	6.76±0.33c	7.42±0.43ab	7.56±0.17a	6.35±0.20A	7.04±0.00A	6.87±0.08A	7.14±0.15A	6.68±1.44A
13	Benzaldehyde	000100-52-7	0.44±0.17ab	0.20±0.01b	0.38±0.05ab	1.38±1.05a	1.04±0.61ab	0.42±0.23B	0.34±0.14B	0.26±0.11B	0.26±0.08B	1.21±0.84A
14	Octanal	000124-13-0	0.87±0.19c	1.12±0.02bc	1.97±0.25a	1.56±0.20ab	1.87±0.51a	1.79±0.17A	1.48±0.13AB	1.60±0.20AB	1.43±0.27B	1.61±0.05AB
15	2,4-Heptadienal, (E,E)-	004313-03-5	0.69±0.09a	0.70±0.01a	0.60±0.01ab	0.48±0.03b	0.51±0.14b	0.31±0.12C	0.62±0.22AB	0.71±0.12AB	0.40±0.14BC	0.77±0.23A
16	2-Tridecenal, (E)-	007069-41-2	1.33±0.04a	1.24±0.04ab	1.05±0.06c	1.15±0.05bc	1.27±0.16ab	1.32±0.07BC	1.18±0.10CD	1.34±0.10AB	1.05±0.02D	1.49±0.11A
17	Nonanal	000124-19-6	4.44±0.48a	3.83±0.19a	2.86±0.08b	3.99±0.37a	4.13±0.47a	3.89±0.28A	3.77±0.14A	3.93±0.04A	3.35±0.14B	4.01±0.11A
18	2,6-Nonadienal, (E,E)-	017587-33-6	0.45±0.05a	0.47±0.08a	0.37±0.10a	0.35±0.01a	0.37±0.10a	0.51±0.04A	0.39±0.08B	0.51±0.06A	0.35±0.03B	0.52±0.01A
19	2-Nonenal, (E)-	018829-56-6	0.53±0.03a	0.50±0.01a	0.41±0.02a	0.40±0.09b	0.43±0.09a	0.45±0.03A	0.43±0.01A	0.49±0.01A	0.35±0.00B	0.45±0.08A
20	Decanal	000112-31-2	0.71±0.19b	1.75±0.11a	0.62±0.09b	0.84±0.25a	1.04±.21a	0.97±0.33A	1.12±0.50A	0.87±0.12A	0.65±0.03A	1.03±0.07A
21	Benzaldehyde, 4-methoxy-	000123-11-5	0.13±0.04a	0.16±0.01a	0.26±0.01a	0.21±0.04a	0.19±0.04a	0.20±0.04A	0.23±0.07A	0.18±0.03A	0.23±0.04A	0.21±0.06A
22	Undecanal	000112-44-7	0.73±0.05c	0.33±0.03b	0.53±0.06a	0.35±0.06b	0.44±0.06b	0.27±0.03C	0.55±0.14B	0.70±0.03A	0.47±0.03B	0.50±0.10B
23	2,4-Decadienal	002363-88-4	0.19±0.04ab	0.18±0.01b	0.22±0.01a	0.14±0.03b	0.20±0.05a	0.18±0.04AB	0.15±0.01B	0.23±0.03A	0.19±0.01AB	0.25±0.06A
**Ketones**												
24	2-Heptanone	000110-43-0	1.94±0.04b	1.47±0.02a	2.31±0.20ab	1.63±0.28b	1.52±0.18b	1.74±0.31B	2.25±0.06A	1.88±0.12AB	1.71±0.36B	1.75±0.19B
25	2-Heptanone, 6-methyl-	000928-68-7	2.30±0.03a	2.14±0.10a	2.00±0.47a	1.82±0.29ab	1.43±0.29b	1.29±0.16B	1.64±0.05AB	1.52±0.14AB	1.31±0.33B	1.87±0.24A
26	2,3-Octanedione	000585-25-1	5.50±0.51ab	5.72±0.66ab	4.59±0.37c	6.08±0.39a	5.02±0.03bc	4.90±0.75AB	5.67±0.52A	5.57±0.21A	4.36±0.03B	5.23±0.20A
27	2-Octanone	000111-13-7	2.25±0.42a	1.70±0.17b	1.84±0.22ab	1.64±0.21b	1.42±0.34b	1.82±0.12A	1.68±0.01A	1.90±0.03A	1.67±0.27A	1.59±0.24A
28	3-Octen-2-one	001669-44-9	0.72±0.17a	0.66±0.03a	0.71±0.14a	0.56±0.09a	0.61±0.01a	0.71±0.13AB	0.70±0.15AB	0.59±0.11B	0.61±0.02B	0.88±0.18A
29	3,5-Octadien-2-one	038284-27-4	4.15±0.24b	4.96±0.39a	3.98±0.33b	4.89±0.46a	4.19±0.45b	4.57±0.02AB	4.73±0.80AB	5.49±0.13A	4.39±0.01B	4.87±0.99AB
30	(E,E)-3,5-Octadien-2-one	030086-02-3	9.46±0.47a	8.85±0.75a	9.92±0.19a	10.04±0.10a	9.63±1.15a	10.56±1.33AB	10.33±0.54AB	9.65±0.04AB	11.24±1.31A	8.92±0.78B
31	2-Decanone	000693-54-9	2.09±0.50a	1.36±0.10b	1.45±0.15b	1.25±0.05b	1.41±0.22b	1.60±0.40AB	1.38±0.06ABC	1.66±0.09A	1.31±0.11BC	1.15±0.02C
32	Carvone	000099-49-0	0.44±0.04a	0.17±0.00b	0.26±0.12b	0.23±0.02b	0.26±0.14b	0.68±0.34A	0.35±0.05B	0.30±0.00B	0.22±0.00B	0.39±0.02B
33	2-Undecanone	000112-12-9	2.93±0.31a	2.74±0.18a	2.64±0.22a	2.93±0.09a	2.63±0.15a	2.75±0.47AB	2.61±0.07B	3.18±0.22A	3.08±0.35AB	2.89±0.19AB
34	3-Decen-2-one	010519-33-2	1.88±0.26a	1.26±0.04c	1.72±0.10ab	1.54±0.01b	1.73±0.11ab	1.69±0.17C	1.53±0.01D	1.92±0.03B	1.71±0.03C	2.11±0.07A
35	Cyclopentanone, 3-methyl-2-pentyl-	1000131-95-1	0.87±0.06a	0.69±0.04c	0.72±0.03bc	0.89±0.01a	0.76±0.01b	0.92±0.01A	0.78±0.03B	0.94±0.00A	0.76±0.08B	0.93±0.06A
36	5,9-Undecadien-2-one, 6,10-dimethyl-	000689-67-8	0.29±0.10a	0.27±0.01ab	0.24±0.04ab	0.20±0.01b	0.22±0.01ab	0.23±0.01C	0.21±0.01C	0.33±0.02A	0.17±0.03D	0.28±0.02B
**Acids**												
37	Butanoic acid, 4-(dimethylamino)-3-hydroxy-	000542-06-3	13.25±1.16a	11.36±1.00a	14.60±1.11a	6.64±3.56b	12.35±2.78a	13.91±0.73A	10.08±4.14B	10.78±0.86AB	11.92±0.08AB	13.63±0.39A
38	n-Hexadecanoic acid	000057-10-3	0.19±0.07a	0.25±0.03a	0.17±0.03a	0.14±0.06a	0.15±0.11a	0.26±0.05A	0.31±0.02A	0.20±0.00AB	0.11±0.06B	0.27±0.14A
**Sulfocompounds**												
39	Carbamodithioic acid, diethyl-, methyl ester	000686-07-7	0.30±0.04a	0.19±0.00b	0.15±0.04b	0.05±0.01c	0.05±0.01c	0.27±0.09B	0.23±0.00B	0.43±0.11A	0.25±0.11B	0.19±0.04B

Data are mean ± standard deviation (n = 3). Values in rows with different lowercase and uppercase letters represent significant differences in the ammonia and salinity treatments, respectively ($p < 0.05$).
